# An unusual case of a traumatic splenic rupture masquerading as myocardial ischemia: a case report

**DOI:** 10.1186/s12245-022-00469-7

**Published:** 2022-11-17

**Authors:** B. M. Munasinghe, U. P. M. Fernando, Thileep Kumar, Chamika Huruggamuwa, K. A. R. L. Kuruppu, G. G. C. Hewawasam

**Affiliations:** 1 Department of Anaesthesiology and Intensive care, District General Hospital, Mannar, Sri Lanka; 2 Department of Anaesthetics, Queen Elizabeth the Queen Mother Hospital, Margate, UK; 3 National Blood Transfusion Service, Colombo, Sri Lanka; 4 Department of Surgery, District General Hospital, Mannar, Sri Lanka; 5 Wrexham Maelor Hospital, Wrexham, Wales

**Keywords:** Splenic injury, Traumatic splenic rupture, Life-threatening haemorrhage, Case report

## Abstract

**Background:**

The spleen is one of the most frequently injured abdominal organs during trauma, which can result in intraperitoneal bleeding of life-threatening magnitude. Although splenic injury secondary to trivial trauma comprises a minor fraction of abdominal injuries, undiagnosed or delayed diagnosis may result in a complicated clinical course.

**Case presentation:**

One such event is presented here, wherein a late diagnosis of an advanced grade splenic injury following a trivial trauma initially presented in disguise as acute myocardial ischaemia in a previously healthy South Asian woman in her late 30s. Emergency laparotomy and splenectomy were performed with simultaneous massive transfusion for a 3.5-L blood loss. She subsequently had an uncomplicated clinical course with regular surgical follow-up.

**Conclusion:**

Splenic injuries might present with ambiguous symptoms such as atypical chest pain and shoulder pain, necessitating attending clinicians to have a high degree of suspicion, especially in busy units such as the emergency department (ED).

## Background

Eighty percent of abdominal trauma cases in the ED involve blunt trauma [1]. Traumatic splenic rupture is seen in approximately 32% of blunt injuries to the abdomen, predominantly following road traffic accidents and falls [2]. Initial presentation following a splenic injury varies from mild left hypochondrial pain, radiating pain to the left shoulder [3], or haemodynamic instability depending on the degree of blood loss. In hypovolaemic patients, anaemia and hypoxia might lead to myocardial ischaemia following advanced grades of splenic injuries. Clinically sinister splenic injuries following relatively minor injuries should be given closer attention, as there could be predisposing microscopic and macroscopic histological abnormalities of the spleen. Herein, we present an unusual case of advanced-grade splenic rupture resulting from a trivial trauma; the patient presented with chest pain radiating to the left shoulder, whereas she was haemodynamically stable on admission. Failure to elucidate the trauma history at the onset and the electrocardiogram (ECG) findings suggestive of inferior ischaemia during the first encounter resulted in acute coronary syndrome management with antiplatelets and anticoagulants. This case report highlights the necessity of excluding more sinister differential diagnoses, such as underlying splenic injuries, in cases of a combination of atypical chest and left shoulder pain and trauma however trivial they may be, which might lead to considerable modifications in subsequent management and resultant favourable outcomes.

## Case presentation

A 39-year-old American Society of Anaesthesiology stage 1, body mass index 23 kg m^-2^, South Asian female presented to the ED of a District General Hospital in Sri Lanka with atypical chest pain and left shoulder pain that had lasted for 3 days. She did not disclose any autonomic features, difficulty breathing, or cough with fever. On examination, she was not pale. Her cardiorespiratory parameters were stable (noninvasive blood pressure, 112/67 mmHg; pulse rate, 88 per minute; peripheral oxygen saturation, 98% on room air; and respiratory rate, 16 per minute). She denied any history of trauma. There was no localized tenderness over the chest. The COVID-19 rapid antigen test result was negative. The ECG showed ‘T’ inversions in inferior leads (lead II, III, and aVF). The troponin I titre was negative. Chest X-ray was normal. She was diagnosed as having unstable angina considering her persistent symptoms. Loading doses of 300 mg oral aspirin, 300 mg clopidogrel, and 40 mg atorvastatin were prescribed along with 60 mg subcutaneous enoxaparin twice a day. Subsequently, the patient was transferred to the medical unit. The repeated ECG did not reveal any dynamic changes. During the ward stay, she complained of persistent symptoms for which sublingual glyceryl trinitrate was administered. The rest of the blood investigations yielded normal results. Eighteen hours after admission, a sudden haemodynamic collapse was witnessed. Her blood pressure dropped to 70/40 mmHg, and her pulse rate increased to 120 beats per minute with a very low volume. She was found to be severely pale. The repeated haemoglobin level was 3.8 g/dl. Immediate resuscitation was commenced with supplemental oxygen. A massive transfusion protocol with transfusion of blood and blood products was initiated. Further history revealed that the patient had had an accidental fall onto a hard surface from an approximate height of 2 feet with impact on her left upper abdomen around the time of onset of the presenting symptoms. An ultrasound scan of the abdomen revealed a splenic laceration with an expanding subscapular haematoma and haemoperitoneum. Following a haematology consultation, 10 mg/kg tranexamic acid and 1 mg/kg protamine sulfate were intravenously administered. Details of the results of the initial and subsequent investigations are illustrated in Table 1.

**Table 1:** Preoperative investigation results

Investigation	Value		Reference
	On admission	Immediately following haemodynamic collapse	
White blood count	10,200	13,000	4-11,000/mm3
Haemoglobin	13.6	3.8	10-14 g/dL
Haematocrit	40.2%	13.1	35-55%
Platelets	254,000	120,000	150-450,000/mm3
Serum Na+	145	142	135-155 meq/L
Serum K+	4.4	4.1	3.5-5.5 meq/L
Blood urea	3.9	4.3	3.3-6.6 mmol/L
Serum creatinine	0.8	0.9	0.7-1.3 mg/dL
Aspartate aminotransferase	25	36	10-35 μ/L
Alanine aminotransferase	28	40	9-55 μ/L
Prothrombin time	10	20	11-13 s
INR	0.9	1.58	0.8-1.1
Activated partial thromboplastin time	33	64	30-40 s
Serum amylase	50	55	40-140 μ/L
Serum bilirubin	0.9	1.0	<1.2 mg/dL
Arterial blood gas analysis	Not performed	pH 7.20	7.35-7.45
		PCO_2_ 36	35-45 mmHg
		PO_2_ 250 (O_2_ 15 L/min)	80-100 mmHg (room air)
		HCO3^-^ 16	22-26 meq/L
		Base excess -10	-2 to +2
		Lactate 6.6	<2mmol/L

**Fig. 1:**
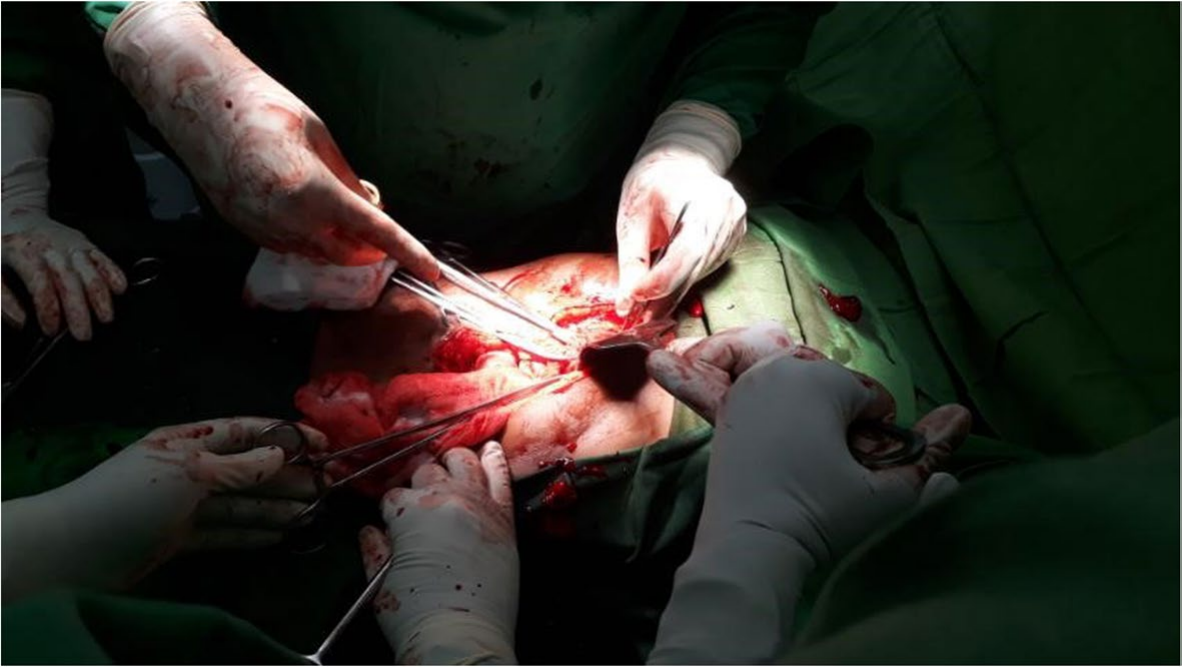
Emergency exploratory laparotomy being performed

**Fig. 2:**
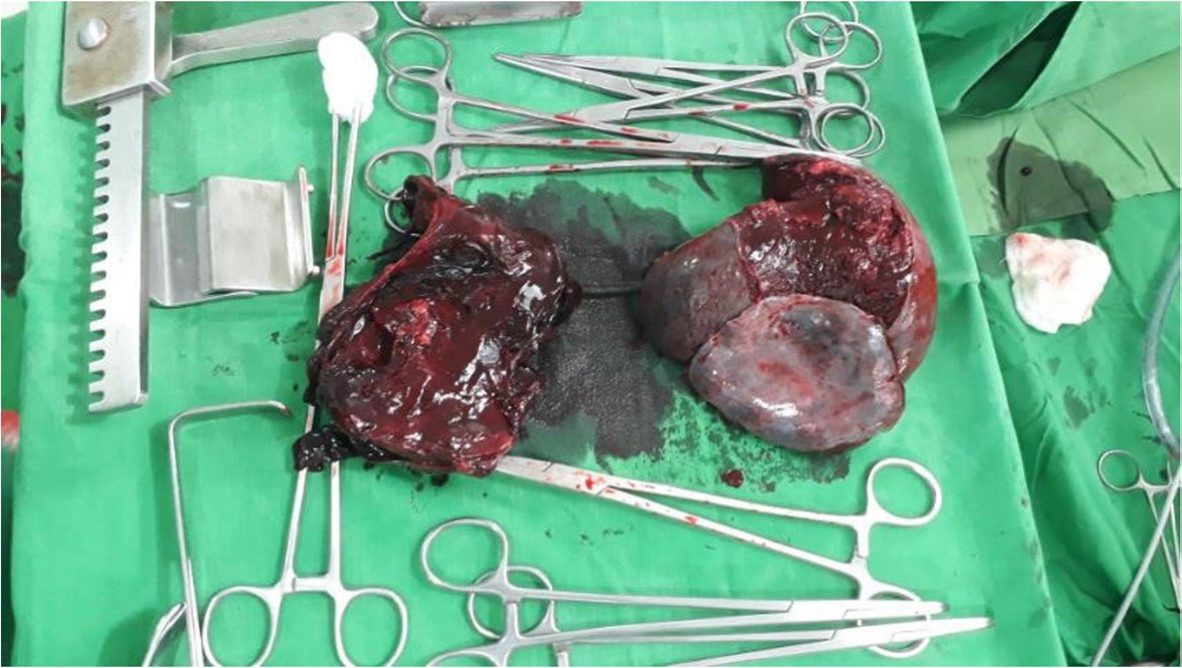
Macroscopic appearance of the spleen following splenectomy

The patient was taken to the operating theatre for emergency exploratory laparotomy. On induction, her blood pressure was 92/65 mmHg, and her pulse rate was 100 beats per minute. She was induced with intravenous ketamine 50 mg, midazolam 1 mg, and fentanyl 75 μg. During the surgery, two linear lacerations extending from the capsule to the hilum were detected in the middle part of the spleen with a large haematoma, indicating an American Association for the Surgery of Trauma (AAST) grade 4 splenic injury with an estimated progressive blood loss of 3.5 L. An urgent splenectomy was warranted with an ongoing massive blood transfusion. The spleen was found to be congested, with an approximate size of 10 cm × 6 cm × 5 cm (Fig. 1. Performance of emergency exploratory laparotomy; Fig. 2. Macroscopic appearance of the spleen following splenectomy).

The massive transfusion was continued, requiring 6 units of packed red cells, 600 mL of fresh frozen plasma, 20 units of cryoprecipitate, and an adult pool of platelets. There was no other visceral organ injury. Haemostasis was achieved, and the patient was admitted to the intensive care unit. Clotting parameters were assessed by the prothrombin time, activated partial thromboplastin time, and platelet count due to the unavailability of point-of-care testing. All tests yielded normal results. Ten hours later, with stable haemodynamics and normalized arterial blood gas analysis, the trachea was extubated. Her ECG changes reverted back to normal within 24 h. On postoperative day two, she was transferred back to the ward. The 2D echocardiogram did not reveal regional wall motion abnormalities. A coronary angiogram was arranged on an outpatient basis after 2 weeks, which revealed a normal coronary vasculature. The lipid profile and fasting blood sugar levels were normal.

The patient was regularly followed up in the surgical clinic, with pneumococcal, meningococcal, and *Haemophilus influenza* b vaccinations arranged at the 2-week follow-up. At the 1-year follow-up, she had been devoid of any life-threatening sepsis or any other surgery-related complications. Following the incident, ED doctors were briefed on the sequence of events. The necessity of a complete history and examination with increased vigilance during trauma was reiterated, and the exclusion of probable differential diagnoses by relevant history, clinical examination, and utility of subsidiary investigations such as bedside focused assessment with sonography in trauma (FAST), whenever appropriate, was encouraged, especially in presentations with atypical chest pain.

## Discussion

The spleen, the largest organ in the lymphatic system, lies between the 9th and 11th ribs in the left hypochondrium [4]. It is considered the most frequently damaged organ following blunt trauma to the abdomen [5]. Its soft consistency, sinusoidal structure, fragile capsule, and highly vascular nature make it more prone to injury with the potential for life-threatening internal haemorrhage [6].

Patients may present with varying degrees of symptomatology following splenic injury. Left hypochondrial and pleuritic chest pain are common [7], while a referred type of left shoulder pain, called Kehr’s sign, is found in approximately 20% of patients [2]. Features of peritonitis and hypovolaemia may be evident in patients with internal bleeding. A FAST might reveal splenic injury with intraperitoneal bleeding. Computed tomography (CT) is considered the gold standard imaging modality with increased sensitivity and specificity. Furthermore, it aids in staging the severity of the injury, which might be directly managed in either operative or nonoperative pathways [8]. ECG changes associated with splenic injury could be the result of multiple factors. Hypovolaemia and tachycardia leading to impaired myocardial perfusion/demand balance and associated myocardial contusions, especially in the background of concurrent chest injuries, could result in varying degrees of such changes [9]. It is imperative to resuscitate without delay and look for chest injuries in such instances.

In the literature, anticoagulants and antiplatelets have been implicated as causative factors of atraumatic splenic rupture [10–13]. Furthermore, the outcome following splenic trauma in patients who were already on anticoagulants was found to be worse in a study by Bhattacharya et al. [14]. By careful analysis of the sequence of events in our case, it is possible that the patient acquired a minor degree of splenic injury during the trauma, and the grade of injury was advanced by concurrent therapy with dual antiplatelets and low molecular weight heparin due to clot dislodgment or systemic anticoagulatory effect. The possible mechanism of the patient’s initial ECG is unclear, as she was haemodynamically stable on presentation, and the subsequent cardiac assessment was normal. We came across a very similar case of a ruptured spleen needing exploratory laparotomy reported by Allan et al. 20 years ago [15]. The authors identified similar ECG changes in an elderly patient with known coronary artery disease who initially presented with ischaemic-type chest pain and for whom unstable angina was subsequently managed with IV heparin. Persistent haemodynamic instability despite fluid therapy and worsening left hypochondrial pain warranted a CT scan of the abdomen, which revealed features suggestive of splenic injury and gross haemoperitoneum, necessitating urgent laparotomy. Similar to our case, there had been a trivial injury not disclosed during the initial medical assessment.

Had the patient disclosed this trauma, there could have been the possibility of detection of lower grade splenic injury and avoidance of antiplatelet and anticoagulant therapy. Considering the initial haemodynamic stability, transferring her to a nearby tertiary care centre for a CT scan with contrast studies (which was unavailable in our centre) was a possibility to further delineate the degree of splenic injury. Nonoperative management could have been exercised on account of the haemodynamic stability and CT findings. Whether there had been a delayed rupture of the spleen precipitated by medical therapy could not be excluded due to the unavailability of advanced histological studies and staining techniques in our institution.

The patient presented to our institution around the first wave of the COVID-19 pandemic in Sri Lanka, where the perceived fear of acquiring the infection was noticeable among the public. The effects of COVID-19 on health-care-seeking behaviour, the overlooking of non-COVID-related cases, the distancing of patient-physician contact as a result of telecommunication as well as the use of personal protective equipment, and the resultant strain on health care workers have already been discussed in the literature [16], which may have been contributory factors in this clinical scenario. It is thus imperative to encourage patients to disclose relevant details pertaining to their presentation. Performing in-depth situational analyses in cases of misdiagnosis or treatment, creating a feedback system enforcing the correct decision-making process, and setting up local guidelines are pivotal in such instances.

## Conclusion

Blunt abdominal trauma may present initially with ambiguous clinical signs that mask any serious internal abdominal injury, thereby making the diagnosis more challenging. Exercising a high degree of suspicion may be the only key to setting the clinical management on the right trajectory. Splenic injury is notorious for causing detrimental clinical outcomes when misdiagnosed. As in the illustrated case, when a high-grade injury ensues secondary to trauma, the same downwards spiral could follow if clinicians do not intervene in a timely manner. The importance of thorough history taking and guided physical examination using bedside testing, such as FAST, cannot be overemphasized to delineate the working diagnosis, especially in the ED setup, to avoid erroneous management.

## Data Availability

Data sharing is not applicable to this article as no datasets were generated or analyzed during the current study.

## Abbreviations

ECG: Electrocardiogram
ED: Emergency department
AAST: American Association for the Surgery of Trauma
FAST: Focused assessment with sonography in trauma
CT: Computed tomography
